# Association of Plasma Renin Activity with Risk of Late Hypertension in Pediatric Patients with Early Aortic Coarctation Repair: A Retrospective Study

**DOI:** 10.3390/life15040656

**Published:** 2025-04-16

**Authors:** Irina-Maria Margarint, Tammam Youssef, Iulian Rotaru, Alexandru Popescu, Olguta Untaru, Cristina Filip, Ovidiu Stiru, Ancuta-Alina Constantin, Vlad Anton Iliescu, Radu Vladareanu

**Affiliations:** 1 Faculty of Medicine, Carol Davila University of Medicine and Pharmacy, 050474 Bucharest, Romania; irina-maria.margarint@drd.umfcd.ro (I.-M.M.); cristina.filip@umfcd.ro (C.F.); ovidiu.stiru@umfcd.ro (O.S.); ancuta-alina.constantin@umfcd.ro (A.-A.C.); vladanton.iliescu@gmail.com (V.A.I.); vladareanu@gmail.com (R.V.); 2 Department of Cardiac Surgery, Emergency Clinical Hospital for Children “Maria Skłodowska Curie”, 077120 Bucharest, Romania; rotaru.iulian7@yahoo.com (I.R.); alexandru.a.g.popescu@gmail.com (A.P.); untaru_olguta@yahoo.com (O.U.)

**Keywords:** aortic coarctation, vascular dysfunction, renin–angiotensin system, hypertension, neonates

## Abstract

Background: Coarctation of the aorta (CoA) represents 5% to 7% of all congenital heart diseases. Surgery and interventional methods offer great short-term results, but the occurrence of postoperative hypertension associated with cardiovascular and cerebral vascular disease increases mortality and morbidity in the long term. This study aims to investigate risk factors associated with postoperative hypertension in pediatric patients with early repair of isolated aortic coarctation. Subjects and Methods: A total of 41 patients with isolated aortic coarctation were included. The mean age was 35.3 ± 46.34 days. Early repair under one month was performed in 65.9% of patients. In all except two patients, end-to-end anastomosis was used. A follow-up at two years revealed an incidence of 58.5% of hypertension. Using logistic regression, preoperative renin plasma concentration above the upper normal level (46.1 μUI/mL) was independently associated with the occurrence of hypertension (OR = 2.49, 95% CI = 2.001–5.03, *p* = 0.001). Conclusion: Coarctation of the aorta is not just a simple mechanical obstruction of the aorta and should be seen and managed as a systemic disease. Abnormal preoperative renin concentrations were independently associated with the occurrence of HT at follow-up, suggesting that vascular dysfunction could play a role in hypertension development after successful CoA repair, negatively influencing the long-term prognostic of these patients.

## 1. Introduction

Coarctation of the aorta (CoA) represents 5% to 7% of all congenital heart diseases. Although surgical and interventional treatments have been developed and offer great short-term results, long-term mortality and morbidity are subjects of debate. Several studies report increased mortality and morbidity later in life, related to the occurrence of arterial hypertension (HT). In turn, HT increases the risk of coronary artery disease, heart failure, and cerebrovascular disease and is associated with premature death [1,2].

Early repair of CoA, especially under one year of life, and extended end-to-end anastomosis are reported to reduce the risk of development of HT later in life [3,4,5,6,7,8], while vascular dysfunction and inflammatory status are mechanisms postulated to explain, at least in part, the occurrence of HT, since CoA is considered to be a systemic disease [9,10].

The renin–angiotensin system plays a significant part in the etiology of vascular dysfunction in these patients, and several studies report increased preoperative and postoperative levels with resulting HT due to hypervolemia [11,12]. Elevated proinflammatory cytokines reported in patients with CoA are reported to influence vascular reactivity and upregulate the atheromatous process [13,14,15,16,17,18,19,20,21,22].

The aim of the study was to investigate the association of possible serum biomarkers related to vascular dysfunction, inflammation, and postoperative HT in patients with CoA repair under one year of age.

## 2. Subjects and Method

Between January 2018 and December 2022, 72 patients were transferred to our center (Emergency Clinical Hospital for Children “Maria Skłodowska Curie”, Bucharest, Romania) for severe CoA. The diagnosis of CoA was based on initial clinical suspicion and confirmed by transthoracic echocardiography.

Demographic and clinical characteristics were collected from the medical records and electronic health system.

Indications for CoA repair were made according to The Society of Thoracic Surgeons Clinical Practice Guidelines on the Management of Neonates and Infants with Coarctation [23].

The inclusion criteria were neonates and infants with clinical and echocardiographic diagnosis of severe aortic coarctation.

We defined the following exclusion criteria: (1) age greater than 1 year at surgery time; (2) non-significant aortic coarctation based on clinical exam and TTE; (3) other significant congenital associated heart defects (other than ASD, VSD, aortic bicuspid valve, and non-severe mitral disease); (4) surgical contraindications, severe hemorrhagic diathesis, intractable septic shock, severe comorbidities with very poor prognosis in short term; and (5) other non-cardiac pathologies witch determine arterial hypertension (e.g., renal disease).

Transthoracic echocardiography (TTE) was used for diagnosis and follow-up. The suprasternal notch view allowed for the evaluation of both the anatomy and measurement of dimensions and Doppler-based gradients across the CoA. We used a color Doppler to visualize the turbulence due to the stenosis and pulse, and a continuous wave Doppler to assess peak velocity and flow characteristics in the descending aorta with a diastolic run-off being specific for severe CoA. A hypoplastic arch was defined if the z-score of the diameter was less than −2.0. A Doppler color flow was used to localize the site of obstruction. We used continuous-wave Doppler measurements by measuring the peak instantaneous gradient across the narrowed segment to assess the hemodynamic severity of the lesions. We also considered a peak gradient above 20 mmHg at the isthmus level associated with “runoff” flow and a narrowing of more than 50% compared to the median normal diameter for sex and BMI (or a Z-score < −3 DS) at the level of the aortic isthmus as signs of significant CoA. For the echocardiography assessment, Philips EPIQ CVX Cardiology Ultrasound System was used.

Cardiac computed tomography (CT) was used to evaluate the entire aorta and its branches, collateral mapping, and the surrounding vascular and extracardiac structures.

Preoperative plasma concentrations of the following biomarkers were measured after an overnight fast of more than ten hours: renin, aldosterone, interleukin-6 (IL-6), and tumor necrosis factor (TNF). We defined the “normal intervals” of the following biomarkers, according to the values provided by our laboratory: renin between 4.4 and 46.1 μUI/mL measured in the supine position, aldosterone in the supine position between 2.21 and 35.3 ng/dL, normal IL6 value below 7 pg/mL, and normal value of TNF below 8.1 pg/mL.

The surgical technique consisted of an extended end-to-end anastomosis performed via a left thoracotomy. In two patients, a residual gradient of 30 mmHg was noted, and Doppler ultrasound revealed turbulent flow with persistent diastolic flow at the level of the distal arch. Given these findings, a decision was made to perform a reversed subclavian flap angioplasty.

Patients were followed up at 2 years, with clinical evaluations that included a TTE and repeated measurements of blood pressure in the right upper limb in an ambulatory setting using Philips Intelivue MP30 Monitor (Boblingen, Germany) (using automatic measurement with a small pediatric cuff adapted for arm length). Postoperative blood pressure was monitored at 3, 6, 12, and 24 months. Blood pressure was measured in the right upper limb during scheduled follow-up visits at 3, 6, 12, and 24 months postoperatively, using an automated oscillometric monitor (Philips Intellivue MP30) with pediatric cuffs appropriately adapted to arm length and circumference. All measurements were performed in a calm setting, with the patient in a resting supine position for at least five minutes before recording. To ensure accuracy and reproducibility, three consecutive measurements were obtained at 15 min intervals during each visit. The mean value of these readings was used for clinical evaluation. The same monitoring device was used consistently across all time points for all patients. Hypertension was diagnosed according to the 2017 American Academy of Pediatrics (AAP) Clinical Practice Guidelines, defined as: systolic and/or diastolic blood pressure ≥ the 95th percentile for age, sex, and height on at least two separate follow-up visits, or the presence of normal blood pressure values while receiving antihypertensive medication. Percentile thresholds were based on standard normative tables provided in the AAP guidelines, which account for age-, sex-, and height-specific reference values. If all three results exceeded the above threshold during the follow-up period, the patient was considered to have HT.

### Statistical Analysis

Statistical analysis was conducted with Wizard 2 Statistical Software for Mac OS (Wizard–Statistics & Analysis^®^, Raipur, Chattisgarh, India). Summary statistics are presented as absolute numbers and percentages for categorical values and as the mean and standard deviation for continuous values.

The incidence of postoperative HT was investigated as the primary outcome. In order to investigate preoperative factors associated with the development of HT after CoA repair, multivariable analysis was performed using logistic regression and taking into account a model that included variables achieving a *p*-value < 0.1 in univariate analysis. A predictive modelling strategy with the backward stepwise method of entering data was then used. Variables included in the univariate analysis were the following: age, premature status, severe and moderate left ventricular dysfunction, bicuspid aortic valve, crenel, gothic and roman aortic arch, the diameter of the distal arch and at the level of the isthmus, doppler peak gradient across the isthmus, doppler velocity at the isthmus, doppler velocity at the level of the abdominal aorta (measured at diaphragm level), systolic pressure gradient between the upper and lower limbs, necessity of inotropic support, serum concentrations of renin, aldosterone, IL6 and TNF above the normal thresholds, and upper and lower limb serum lactate. Logistic regression results are presented as odds ratios (OR) with confidence limits and *p*-values.

## 3. Results

**Figure 1:**
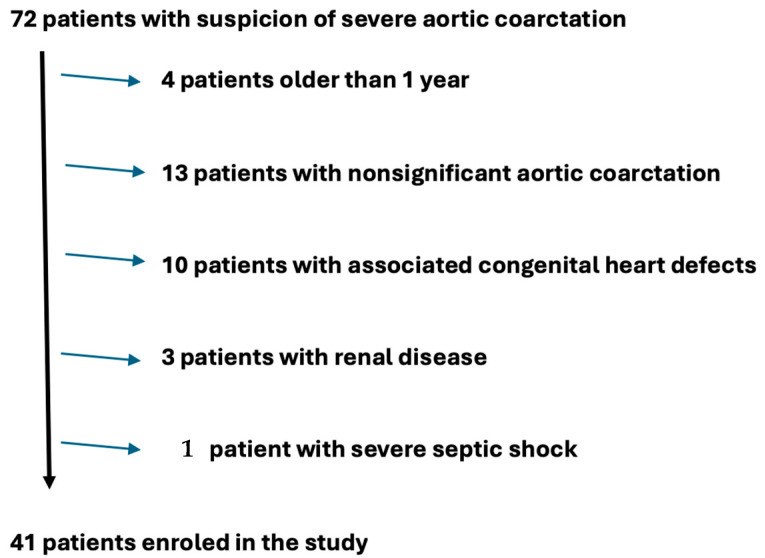
The total number of patients included in the study after applying exclusion criteria.

After the exclusion criteria were applied, 41 patients were enrolled in the study. A total of 31 patients were excluded (4 patients older than 1 year, 13 patients with nonsignificant aortic coarctation, 10 patients with associated congenital heart defects, 3 patients with renal disease, and 1 patient with severe septic shock) (Figure 1).

Clinical symptoms and signs in neonates with early presentation included the following: tachypnea and difficulty feeding/failure to thrive, reduced pulse oximetry in the lower limbs compared to upper limbs, diminished femoral pulses, a blood pressure gradient between the upper and lower extremities (systolic > 20 mmHg), oliguria and renal failure, abdominal bloating, interscapular systolic murmur, and, eventually, signs associated with other congenital heart lesions such as aortic stenosis or ventricular septal defect.

Patient characteristics are shown in Table 1. The mean age at the time of surgery was 35.3 ± 46.3 days, with 65.9% of patients being younger than one month and 9.8% classified as preterm neonates. Only five patients (12.1%) presented with severe left ventricular dysfunction (LVEF < 25%). The incidence of HT at one year after surgery was 58.5%. Patients with HT had a significantly higher incidence of severe left ventricular dysfunction, crenel, and gothic arch when compared to those without HT (Table 2).

No perioperative deaths were recorded, and one case of chylothorax and one case of superficial wound infection were documented. At the 2-year follow-up, all patients were alive, and no re-coarctation was documented.

### 3.1. Coarctation of the Aorta—Characteristics

The incidence of particular arch anatomy was the following: the most frequent form was the Roman shape type in 46.3% of cases, followed by the Gothic type in 17.1% of cases, and the Crenel type in 9.8% of patients. The aortic arch’s diameter was 5.84 ± 1.17 mm at the proximal level, 4.92 ± 1.13 mm at the distal level, and 2.72 ± 0.92 mm at the isthmus level. The systolic pressure gradient between the upper and lower limbs was 33.67 ± 16.85 mmHg. A transthoracic Doppler study of the coarctation area showed a 52.7 ± 19.37 mmHg peak gradient and a velocity of 3.15 ± 1.2 m/s. Velocity at the level of the abdominal aorta was 0.49 ± 0.23 m/s.

The mean duration of surgery was 110 ± 28.56 min, and the mean aortic clamp time was 25.97 ± 9.97 min. Two patients necessitated reclamping of the aorta and the enlargement of the aortic arch with the reversed subclavian flap technique after initial extended end-to-end anastomosis because of high gradients measured at the conclusion of the anastomosis. The postoperative Doppler study results were the following: mean maximum velocity at the isthmus was 1.59 ± 0.5 m/s, mean maximum gradient across the isthmus was 16.68 ± 7.27 mmHg, while mean maximum velocity at the level of the abdominal aorta was 0.83 ± 0.23 m/s. The mean pressure gradient between the upper and lower limbs was 7.31 ± 4.26 mmHg.

### 3.2. Preoperative Biomarkers

The mean renin plasma concentration was 57.1 ± 42.27 μUI/mL, and more than half of the patients (67.5%) had a plasma renin concentration above the normal upper limit (46.1 μUI/mL). The mean aldosterone plasma levels were 94.89 ± 103.21 ng/dL, IL6 had a mean plasma concentration of 4.25 ± 2.27 pg/mL, and TNF had a mean plasma concentration of 6.76 ± 7.68 pg/mL. Except for renin (72.15 ± 23.61 μUI/mL vs. 35.18 ± 18.2 μUI/mL; *p* = 0.02), and the incidence of renin levels above the upper limit (95.83% vs. 17.64%, *p* = 0.04), no significant differences were noticed in preoperatively biomarker levels in post-surgical normotensive versus hypertensive patients.

### 3.3. Logistic Regression

Table 3 shows the results of the univariate analysis for the variables that have achieved a *p*-value < 0.1. Severe left ventricle dysfunction (OR = 3.13, 95% CI = 1.27–4.39, *p* = 0.02) and gothic arch (OR = 4.21, 95% CI = 2.06–6.29, *p* = 0.01) were included in the final model after backward selection. Plasma renin concentration above the normal upper limit (46.1 μUI/mL) was associated with HT in univariate analysis (OR = 3.35, 95% CI = 2.09–5.39, *p* = 0.04) and, after model adjustment, was an independent factor associated with HT (OR = 2.49, 95% CI = 2.001–5.03, *p* = 0.001).

## 4. Discussion

This study shows that the incidence of HT after successful CoA repair was 58.5% and that high serum levels of plasma renin were independently associated with the development of HT on a two-year follow-up.

Although surgical treatment or transcatheter techniques offer excellent short-term results, long-term morbidity and mortality remain higher in this group of patients [1,2]. Systemic hypertension (HT) is commonly reported at follow-up and can persist even if the aortic obstruction is relieved [24,25,26,27]. HT increases the risk of early coronary artery disease, left ventricular hypertrophy, heart failure, and cerebrovascular events [1,2]. Multiple studies report that postoperative HT is an independent risk factor for premature death [5,28,29,30].

The incidence of HT after CoA repair is up to 47.3% [2]. Multiple studies report that age at the time of the initial repair is the most important predictor of long-term survival and that CoA should undergo repair even sooner than 1 year if HT is present [3,4,5,6]. Lillitos et al. reported that the risk of developing postoperative HT was lowest in neonates and increased 10-fold if CoA was treated in childhood [3]. In our study, we report a high incidence of HT, even in early surgical correction during the first year of life (58.5%). Similarly, Seirafi et al. reported that age at repair was a significant predictor of late HT. They communicated a low, 4.2% incidence of late HT in the group operated under 1 year, and a sixfold increase in late HT, when compared to the group operated after 1 year [4]. A slightly higher age at repair (1.5 years) was communicated by Brower et al., who also found that late age of repair is associated with late HT and premature death [5]. However, Rinnstrom et al. and Bambul et al., in recent studies, report that the beneficial effect of early repair is reduced over time and that the age of intervention is less important than the age at follow-up [31,32]. Considering age at follow-up, Sendzikaite et al. reported a 46.7% incidence of HT 8.5 years after CoA repair [33], with a lower rate communicated by Brown et al. [34] at 15 years follow-up. Choudhary et al., report an incidence of 44% of HT in patients with an age of repair below 5 years, and that long-term morbidity, especially after 60 years, is largely related to late HT in 140 patients [35].

In our study, the mean age was 35.3 ± 46.34 days, with 65.9% of patients being operated before one month of age. However, we could not confirm the generally accepted theory that the incidence of HT is reduced with CoA repair at a younger age. A possible explanation could be the short follow-up period, considering that some studies have a follow-up period of more than two years. Also, another factor could be the method for blood pressure measurement. While we have performed three separate measurements with the same device, there is a heterogeneity of determining HT in different studies, including 24 h BP measurement, no standardization of type of BP devices, and not taking into consideration that different classes of medication can influence the BP measurement. Also, BP phenotypes (2016 European Society of Hypertension Guidelines) [23] were not categorized, considering that some studies suggest that systolic HT is the dominant phenotype in CoA patients [2,23,36,37,38]. Early repair (65.9% had CoA repair under one month) and extended end-to-end anastomosis (all patients except two cases) did not seem to reduce the incidence of HT in our study; we can speculate that neonatal coarctation is regularly a more severe form (critical/duct-dependent) and in these patients, the benefit of early correction balances the deleterious effect of possible renal ischemia or severe HT through the inability to develop collaterals in such a short time.

Multiple studies suggest that CoA is more than just a narrowing of the aorta, and some studies characterize this pathology as a systemic one with vascular dysfunction, structural modifications of the aortic wall (increased aortic stiffness), and inflammatory mechanisms that promote late HT even after CoA repair.

Vascular dysfunction is an important mechanism in the pathology of HT in patients treated for CoA.

Divitiis et al. reported increased vascular stiffness and abnormal responses to flow in the arteries in the upper part of the body based on pulse wave velocity [39]. Trojnarska et al. also report in a study of 85 patients that CoA is a systemic vascular disorder that leads to progressive vascular and end-organ damage based on flow-mediated dilatation study, intima-media thickness, and pulse wave velocity [40]. Both studies report reduced endothelium arterial relaxation and nitroglycerine-mediated vasodilatation in the brachial artery, reflecting a dysfunction of the capacity of smooth vascular muscle cells to relax. Furthermore, higher intima-media thickness was observed in these patients, which may suggest a congenital side of the vascular changes. Several studies suggest that vascular remodeling after CoA repair and impaired elasticity (increased aortic stiffness) is independent of HT and that abnormalities of the arterial wall led to HT. In turn, HT contributes to vascular changes, creating a vicious circle in these patients [9,10,41]. Table 4 summarizes the main studies investigating impaired aortic elasticity. Ou P et al. reported increased central aortic distensibility, compliance, and increased stiffness based on the beta index in normotensive children with CoA repair [42]. Similar results were reported by Rog B et al. [43] and Shang Q et al. [44], all reporting increased aortic stiffness after successful CoA repair during childhood. Cetiner N et al. [45] demonstrated an increased stiffness index of the abdominal aorta and a decreased flow-mediated dilatation of the brachial artery in patients with coarctation repair in childhood than in controls, suggesting that these changes affect not only the aorta at a central level, but also the peripheral vessel, and increase the cardiovascular mortality and morbidity. The geometry of the aortic arch seems to contribute to increased vascular stiffness, with the gothic arch being linked to increased central aortic stiffness [46,47]. Other authors investigated if different strategies of CoA repair influence the pathological modifications of the aorta wall. Shafer M. et al. [48] reported that ascending aorta stiffness is greater in children treated by surgery of balloon angioplasty, while those with aortic stenting had no significant increase in stiffness compared to the control group. However, Pieper T et al. [49] did not confirm these results in adults treated in childhood.

Preoperative abnormalities in the renin–angiotensin system are reported to induce vascular dysfunction in these patients and promote HT [11,12]. Park et al. demonstrated higher preoperative levels of plasma renin activity [11] and postulated that decreased blood flow to the kidneys increases renin production by the juxtaglomerular apparatus, in turn increasing angiotensin I and II with vasoconstriction and aldosterone secretion. The resulting sodium and water retention expands the extracellular volume and the blood flow to the kidneys. HT is the result of hypervolemia. In another study, Parker et al. reported that renin levels were high even after CoA repair and stated that the renin–angiotensin system plays an important role in the development of postoperative HT [50]. These results were confirmed by Alpert et al., who demonstrated increased PRA in 12 patients with CoA with a sodium-restricted diet in comparison with 8 patients with essential hypertension and 13 control subjects [51]. Lardoux et al. used saralasine, a renin–angiotensin system antagonist, in seven children with postoperative HT and communicated a significant reduction in blood pressure. He stated that an important mechanism in postoperative HT after CoA repair is due to the single kidney Goldblatt model [52]. The single kidney model, as a mechanism for HT, is also sustained in patients with CoA by Alpert et al., who reported that urinary aldosterone concentration, plasma volume, and extracell fluid volume were increased in patients with CoA [51]. Also, Bagby et al. confirmed higher levels of renin activity in a dog model with CoA after a sodium-restricted diet [53]. Fallo et al. reported that HT in CoA is at least in part renin mediated. He measured plasma renin activity after CoA and reported that even though PRA fell after surgery, patients still presented HT [12]. Our study supports these findings, with renin serum levels being independently associated with the occurrence of HT.

Moreover, patients with repaired CoA develop an inflammatory reaction that affects all vessels and could explain the occurrence of HT [13]. In our study, TNF and IL-6 levels were not associated with the development of HT. In contrast, elevated inflammatory cytokines (TNF, Il-6) are reported by Sharma et al. and are related to functional status in adults with congenital heart disease [14]. Brili et al. investigated the levels of proinflammatory cytokines in normotensive patients after CoA repair and communicated higher levels in this group. Proinflammatory cytokines stimulate the expression of adhesion molecules, which in turn upregulate the atheromatous lesions. They postulated that this could explain the higher incidence of coronary artery disease in these patients [15].

A key limitation of this study is its retrospective design, which inherently limits the ability to control for confounding variables and introduces the risk of incomplete or inconsistent data collection. Because patient information was extracted from existing medical records and electronic databases, the accuracy and completeness of the data relied heavily on prior documentation quality. Another limitation of this study is the relatively low number of patients and the short period of follow-up. Additionally, the study may be subject to selection bias, as inclusion was limited to patients referred to a single tertiary cardiac surgery center. This population likely represents more severe or complex cases of coarctation of the aorta, potentially limiting the generalizability of the findings to all infants with CoA. Moreover, follow-up data were available only for patients who returned for scheduled visits, raising the possibility of attrition bias and underestimation or overestimation of late hypertension prevalence.

Future research should focus on prospective multicenter studies with larger cohorts and extended follow-up periods to better understand the long-term evolution of blood pressure in this population. Additionally, interventional studies are needed to evaluate the efficacy of targeted antihypertensive therapies such as ACE-inhibitors in patients with elevated preoperative renin levels. Lastly, the role of ambulatory blood pressure monitoring and exercise-induced hypertension in the early detection of subclinical vascular dysfunction should be addressed in future pediatric follow-up protocols. Another limitation of the study and possible future research is in investigating myocardial damage biomarkers such as high-sensitivity troponin T and N-terminal pro-B-type natriuretic peptide as possible factors in HT development after CoA repair [54]. Lam YY et al. [55] reported that left ventricular dysfunction is common after successful CoA repair and is related to older age at intervention and increased aortic stiffness. Also, global area strain was reduced in patients with CoA repair in childhood compared to controls, suggesting a subclinical left ventricular dysfunction in this group of patients [56].

## 5. Conclusions

We studied 41 patients with isolated CoA repair under one year of age and found an incidence of 58.5% of postoperative HT at two years. Preoperative renin plasma concentrations were independently associated with the occurrence of HT. These results were in accordance with data from the literature and suggest that the renin–angiotensine mechanism could play an important role in the development of HT in patients with CoA repair at an early age. Two conclusions have to be underlined based on this study: (1) it is necessary to find a standardized and reproducible method to measure blood pressure in infants and small children, taking into consideration difficulties for ambulatory 24 h monitoring in this early age and risks of overestimated HT based only on measurements in the uncomfortable in-hospital environment for the child; and (2) the patient with significant high serum renin levels should possibly be monitored closely after surgery with regular renin level checks and targeted HT medication consisting of ACE- inhibitors. Further studies are necessary for a longer follow-up to better understand the mechanism of residual HT, the most important prognostic factors, and the best treatment for HT, taking into account the presumed mechanism.

## Figures and Tables

**Table 1:** Preoperative, intraoperative, and postoperative characteristics of the patients.

Parameter (Unit)	*n* = 41 (100%)
**Preoperative**	
Age (mean, SD days)	35.3 ± 46.34
Surgery under one month of age (*n*, %)	27 (65.9)
Male sex (*n*, %)	36 (87.8)
Premature (*n*, %)	4 (9.8)
Severe Left Ventricular Dysfunction (*n*, %)	6 (14.63)
Moderate Left Ventricular Dysfunction (*n*, %)	8 (20)
Bicuspid aortic valve (*n*, %)	32 (78)
Crenel type aortic arch (*n*, %)	4 (9.8)
Gothic type aortic arch (*n*, %)	7 (17.1)
Roman type aortic arch (*n*, %)	19 (46.3)
Ascending aorta diameter (*n*, SD mm)	8.05 ± 1.82
Distal aortic arch diameter (mean, SD, mm)	4.92 ± 1.13
Proximal aortic arch diameter (mean, SD mm)	5.84 ± 1.17
Aortic isthmus diameter (mean, SD mm)	2.72 ± 0.92
Peak gradient across the isthmus (mean, SD mmHg)	52.7 ± 19.37
Peak velocity at the isthmus (mean, SD m/s)	3.15 ± 1.2
Peak velocity at the level of the abdominal aorta (mean, SD m/s)	0.49 ± 0.23
Systolic pressure gradient between upper and lower limbs (mean, SD mmHg)	33.67 ± 16.85
Inotropic support (*n*, %)	6 (14.63)
**Biomarkers**	
Renin concentration (mean, SD μUI/mL)	57.1 ± 42.27
Renin concentration > 46.1 μUI/mL (*n*, %)	27 (67.5)
Aldosterone concentration (mean, SD ng/dL)	94.89 ± 103.21
Aldosterone concentration > 35.3 ng/dL (*n*, %)	25 (62.5)
IL6 (mean, SD pg/mL)	4.25 ± 2.27
IL6 concentration > 7 pg/mL (*n*, %)	6 (15)
TNF (mean, SD pg/mL)	6.76 ± 7.68
TNF concentration > 8.1 pg/mL (*n*, %)	11 (27.5)
Upper limb serum lactate (mean, SD mmol/L)	2.8 ± 1.53
Lower limb serum lactate (mean, SD mmol/L)	3.12 ± 1.88
**Intraoperative**	
Duration of surgery (mean, SD min)	110 ± 28.56
Reversed subclavian flap (*n*, %)	2 (4.87)
Aortic clamp time (mean, SD min)	25.97 ± 9.97
**Postoperative**	
Peak velocity at the isthmus (mean, SD m/s)	1.59 ± 0.5
Peak gradient across the isthmus (mean, SD mmHg)	16.68 ± 7.27
Peak velocity at the level of the abdominal aorta (mean, SD m/s)	0.83 ± 0.23
Systolic pressure gradient between upper and lower limbs (mean, SD, mmHg)	7.31 ± 4.26
Hypertension (*n*, %)	24 (58.5)

IL6: Interleukin 6; TNF: tumour necrosis factor.

**Table 2:** Comparison between the patients with and without HT; mean ± SD; *n* (%).

Variable	HT + *N* = 24	HT − *N* = 17	*p*
Severe left ventricular dysfunction (*n*, %)	5 (20.8)	0 (0)	0.04
Renin plasma concentration (mean, SD)	72.15 ± 23.61	35.18 ± 18.2	0.02
Renin concentration > 46.1 μUI/mL (*n*, %)	23 (95.83)	3 (17.64)	0.04
Crenel aortic arch (*n*, %)	4 (16.7)	0 (0)	0.03
Gothic aortic arc (*n*, %)	7 (29.7)	3 (17.6)	0.01

**Table 3:** Factors associated with HT.

	Univariate Analisys			Multivariable Analisys		
	OR	95% CI	*p*	OR	95% CI	*p*
Renin plasma concentration	2.31	2.01–3.74	0.05			
Renin plasma concentration (>46.1 μUI/mL)	3.35	2.09–5.39	0.04	2.49	2.001–5.03	0.001
Severe left ventricle dysfunction	3.13	1.27–4.39	0.02	3.63	1.08–8.35	0.02
Gotic arch	4.21	2.06–6.29	0.01	2.83	1.05–10.45	0.01

**Table 4:** Studies reporting increased arterial stiffness after CoA repair in childhood.

Author [Ref.]	Number of Patients	Conclusion
Ou P [42]	40	Decreased central aortic distensibility and compliance of aorta; increased stiffness
Ou P [47]	55	Angulated Gothic aortic arch is associated with increased systolic wave reflection, as well as increased central aortic stiffness and left ventricular mass index
Donazzan [46]	26	Gothic arch shape is associated with a decreased ascending aorta distensibility with an increased loss of systolic wave amplitude across the aortic arch.
Róg B [43]	58	Increased arterial stiffness occurs in both groups: patients after aortic coarctation repair and patients with bicuspid aortic valve
Shang Q [44]	50	In hypertensive CoA, ascending aortic stifness was increased compared to normotensive CoA and controls
Shafer M [48]	49	The ascending aorta of children following surgical repair or balloon angioplasty demonstrated signs of elevated stiffness, whereas those treated by stent implantation showed no difference in stiffness markers when compared to normal controls.
Pieper T [49]	50	CoA patients after surgery or stent implantation did not show significant difference in aortic elasticity
Çetiner N [45]	20	Vascular wall changes in children and adolescents can be seen even after successful coarctation repair and may progress toward overt atherosclerosis at older ages.

## Data Availability

Data are available upon request.
